# Testing an HPV Vaccine Decision Aid for 27- to 45-Year-Old Adults in the United States: A Randomized Trial

**DOI:** 10.1177/0272989X241305142

**Published:** 2024-12-24

**Authors:** Erika L. Thompson, Justin Luningham, Sarah A. Alkhatib, Jessica Grace, Idara N. Akpan, Ellen M. Daley, Gregory D. Zimet, Christopher W. Wheldon

**Affiliations:** Department of Quantitative and Qualitative Health Sciences, University of Texas School of Public Health San Antonio, University of Texas Health Science Center at San Antonio, San Antonio, TX, USA; Department of Population and Community Health, College of Public Health, University of North Texas Health Science Center, Fort Worth, TX, USA; Department of Population and Community Health, College of Public Health, University of North Texas Health Science Center, Fort Worth, TX, USA; Department of Psychology, College of Science and Engineering, Texas Christian University, Fort Worth, TX, USA; Department of Population and Community Health, College of Public Health, University of North Texas Health Science Center, Fort Worth, TX, USA; Department of Population and Community Health, College of Public Health, University of North Texas Health Science Center, Fort Worth, TX, USA; Department of Social Work, College of Health and Public Service, University of North Texas, Denton, TX, USA; Department of Population and Community Health, College of Public Health, University of North Texas Health Science Center, Fort Worth, TX, USA; College of Public Health, University of South Florida, Tampa, FL, USA; Department of Pediatrics, Indiana University School of Medicine and Zimet Research Consulting LLC, Indianapolis, IN, USA; Department of Social & Behavioral Sciences, College of Public Health, Temple University, Philadelphia, PA, USA

**Keywords:** HPV vaccine, decision aid, decision making, adults

## Abstract

**Background:**

In the United States, human papillomavirus (HPV) vaccination among 27- to 45-y-olds (mid-adults) is recommended based on shared clinical decision making with a health care provider. We developed a patient decision aid tool to support the implementation of this mid-adult HPV vaccination guideline. The purpose of this study was to evaluate the effect of a patient decision aid tool for HPV vaccination, HPV DECIDE, compared with an information fact sheet among mid-adults who have not received the HPV vaccine.

**Method:**

Participants were recruited between December 2023 and January 2024. We used a randomized Solomon, 4-group, pretest/posttest design with mid-adults aged 27 to 45 y who were unvaccinated for HPV and balanced based on sex (*n* = 612). The primary outcome was decisional conflict. Intermediate outcomes included knowledge, behavioral expectancies, self-efficacy, and perceived risk. Variables were measured using validated scales. Pretest sensitization was not present; intervention and control groups were compared. Fixed-effects inverse-variance weighting was used to pool effect estimates and determine meta-analytic statistical significance across tests with and without pretest controls.

**Results:**

Participants in the intervention group had significantly lower total decisional conflict scores (B = −3.58, *P* = 0.007) compared with the control group. Compared with the control group, participants in the intervention group showed higher knowledge (B = 0.48, *P* = 0.020), greater intention to receive (B = 0.196, *P* = 0.049) and discuss the HPV vaccine (B = 0.324, *P* ≤ 0.001), and greater self-efficacy about HPV vaccine decision making (B = 3.28, *P* = 0.043). There were no statistically significant results for perceived risks of HPV infection.

**Conclusions:**

The HPV DECIDE tool for mid-adult HPV vaccination shows promise for immediate reductions in decisional conflict and improvement in knowledge, intentions, and self-efficacy about the HPV vaccine. Future studies are warranted to evaluate the effectiveness of this patient decision aid tool in real-world settings.

**Highlights:**

- Shared clinical decision making is recommended for HPV vaccination with mid-adults.
- A patient decision aid for HPV vaccination reduced decisional conflict for mid-adults.
- The HPV vaccine patient decision aid was acceptable to mid-adults.

## Background

The human papillomavirus (HPV) is responsible for causing 6 different types of anogenital and oropharyngeal cancers.^1^ Vaccination serves as an effective preventive measure against these cancers. In 2006, the Advisory Committee on Immunization Practices (ACIP) recommended routine HPV vaccination for adolescent girls aged 11 to 12 y, with catch-up vaccination available for females up to age 26 y. Later, in 2011, the routine recommendation was extended to include adolescent boys aged 11 to 12 y, with catch-up vaccination available for males until age 21 y or up to age 26 y for high-risk groups, such as men who have sex with men and immunocompromised individuals.^2^ More recently, the catch-up age of 26 y was harmonized for males and females.^3^ Despite effective vaccines being readily available, there has been a suboptimal adoption of the HPV vaccine among adolescents, leaving a significant number of adults susceptible to oncogenic HPV infections.^4–6^

In 2018, the US Food and Drug Administration granted approval for the 9-valent HPV vaccine for adults aged 27 to 45 y,^7^ subsequently leading to an updated recommendation from ACIP. In June 2019, ACIP made the decision to recommend the HPV vaccine as a shared clinical decision for individuals aged 27 to 45 y. This recommendation considers not only safety and efficacy but also considers factors such as vaccine effectiveness and cost-effectiveness.^3^ The current guideline for expanded HPV vaccination (i.e., adults aged 27–45 y) suggests that “shared clinical decision-making regarding HPV vaccination is recommended for some adults aged 27 through 45 y who are not adequately vaccinated.” Furthermore, the recommendation advises clinicians to consider discussing HPV vaccination with individuals who are most likely to benefit from it. However, there is a lack of clear guidance on which patients providers should approach for discussions regarding potential vaccination benefits.

Nonetheless, despite guidelines being available, there remains a pressing need for shared decision-making support in clinical practice. The expanded age authorization for the 9-valent HPV vaccine offers an opportunity to reach individuals who were previously considered too old or had exceeded the initial licensure age but who can still benefit from vaccination.^8,9^ While health care providers are aware of the mid-adult vaccination guidelines, there is a substantial gap in their understanding (e.g., which patients to discuss with, key points of the guidelines) for how to effectively translate and implement these recommendations.^10,11^ Hurley et al.^11^ recommended the use of a decision aid to support the shared clinical decision-making process and support physicians in the key talking points from ACIP recommendations. Moreover, the use of a decision aid that is patient facing can help reduce potential downstream inequities. The expanded age option is most likely to benefit populations who are already well-informed about HPV vaccination, aware of the changes to the 9-valent vaccine licensure, and empowered to advocate for their health care choices. Prior research has shown that 27- to 45-y-old US adults with higher educational attainment and engagement with health care settings were more likely to be aware of HPV and HPV vaccination.^12^ Moreover, to assist with decision making, adults in this age range described needing more information about HPV vaccine safety, effectiveness, personal benefit, provider recommendations, side effects, and risks.^13^ Thus, both patients and providers could benefit from decision support tools with this complicated guideline from ACIP. There are limited examples of decision tools for HPV vaccination.^14^ This is likely largely attributable to clear ACIP guidelines for HPV vaccination among adolescents and young adults rather than a shared clinical decision recommendation. As such, any creation of a decision-support tool for mid-adult HPV vaccination should rely on existing standards and frameworks related to decision-making science.

To support patients in shared clinical decision making, we have developed a decision tool^15–17^ that incorporates best practices for shared decision making and international standards for decision tools. The purpose of this study was to determine the effect of the decision aid tool on HPV vaccination decision support outcomes among adults ages 27 to 45 y in the United States who had not been vaccinated. The primary outcome was decisional conflict, and secondary outcomes included intermediate variables related to decision making. Our primary hypothesis was that persons who received the decision tool intervention would have lower decisional conflict compared with those who received the control condition. Exploratory research questions included: What impact did the decision tool have on intermediate outcomes such as knowledge, behavioral expectancies, decisional self-efficacy, and perceived risk? We also described preferences for the implementation of the decision tool from the perspective of end-users.

## Methods

The development of the patient decision aid tool was guided by the Ottawa Decision Support Framework,^16^ which assists in supporting decisions with multiple options. Moreover, the decision aid was constructed in collaboration with community and provider advisory boards. The community advisory board (CAB) consisted of individuals who were unvaccinated for HPV and within the mid-adult age group (27–45 y) targeted for this decision aid. Health care providers made up the practice advisory board (PAB), representing family medicine, internal medicine, and obstetrics/gynecology. The CAB and PAB members were identified through community partners and academic medical centers. The advisory groups met with the research team for 5 rounds to review study materials and provide feedback on the decision aid tool’s content, appearance, and flow. Additional feedback was gained during usability (i.e., content, flow, and visuals feedback; *n* = 10) and field testing (i.e., independent use of the tool with input on future implementation; *n* = 10) with members of the target population. The final phase of tool development included qualitative interviews with health care providers and stakeholders, specifically focusing on the implementation of the tool and its acceptability to medical providers (*n* = 24). During each stage of development, revisions were made incorporating feedback and further refining the decision aid before the study launch.^17^

This study was approved by the North Texas Regional Institutional Review Board. Study participants were recruited through a national survey panel vendor, Centiment.^18^ The inclusion criteria were individuals within the mid-adult age range (27–45 y), unvaccinated for HPV, English speaking, and living in the United States. Individuals who were unsure of their HPV vaccination status were excluded from the study. Participants viewed a consent form prior to the screening survey and indicated consent by proceeding with the survey.

**Figure 1:**
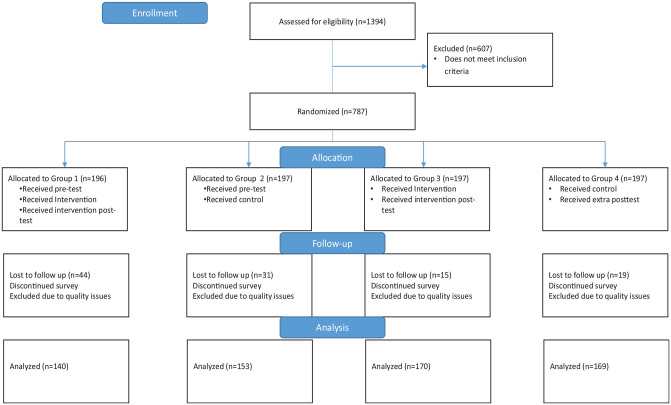
Consort diagram.

A Solomon, 4-group, pretest/posttest research design was used to test the main outcome. In this design, groups 1 and 3 received the intervention, while groups 2 and 4 did not. Groups 1 and 2 received a pretest, whereas groups 3 and 4 did not. This design protects against pretest sensitization in which participants may be “primed” to respond differently on the posttest because of items in the pretest. Screening questions were used to determine eligibility for participation. Individuals determined to be eligible were randomized using urn randomization within the Rivulent platform into 1 of 4 groups (Figure 1). Randomization was also balanced based on sex. Individuals determined to be ineligible during the screening questions were excluded from participation. The intervention group received the Web-based decision tool, HPV DECIDE, which was interactive and provided information on the mid-adult HPV vaccination guideline, the decision, pros and cons of the decisions, values clarification, information dissemination based on values, and a tailored action plan based on the decision. The control group viewed the Centers for Disease Control and Prevention’s Vaccine Information Sheet on HPV vaccination embedded within the survey.^19^

### Sample

Participants were recruited between December 2023 and January 2024. There were 1,394 participants assessed for eligibility, and 787 individuals met inclusion criteria and were randomized into 1 of the 4 groups (Figure 1). Quotas were enabled within the survey to ensure equal distribution of males and females within each group. Participants who did not complete the survey or had quality issues in their responses were removed, leaving between 140 and 170 participants in each group used for analysis. The target sample size was estimated to detect a change in the decisional conflict scale.^20^ Anticipated effect size based on the Cochrane Database Systematic Review of decision aids on decisional conflict subscales ranged from 4.04 to 9.28.^21^ We calculated the variance of these effect sizes to be approximately 270 based on the average sample size (*N*∼ 300) across the studies reviewed. Based on these calculations, a sample size of 350 would enable us to detect effect sizes (i.e., mean differences in the decisional conflict subscales) as small as 3.5. Our analytic sample was 632.

### Measures

Pre- and postsurveys were administered to participants in group 1 (subset of intervention recipients) and group 3 (subset of control recipients). Posttests were administered to all participants. Baseline measures were collected in the pretest survey. The primary outcome was decisional conflict. Secondary outcomes included vaccine decision, intentions, knowledge, expectations, decisional self-efficacy, and perceived risks.

Decisional conflict was measured using the 16-item Decisional Conflict Scale, with response options ranging from *strongly agree* (0) to *strongly disagree* (4); the scores ranged from 0 (*no conflict*) to 100 (*extreme decisional conflict*). The scale assesses an individual’s uncertainty in making decisions related to their health.^20^ Perceived uncertainty can be motivated by insufficient information about available options, lack of clarity on how choices align with personal values, and absence of support to inform decisions.^20^ We evaluated participants’ ability to make decisions about HPV vaccination based on personal values, social influence or pressure, availability of resources, and understanding of risks and benefits. The scale had excellent internal reliability, with the set of all Decisional Conflict posttest items having a Cronbach’s alpha of 0.96 and all subscales resulting in an alpha of 0.89 or 0.90.

Participants were expected to indicate their decision on getting the HPV vaccine. Response options included, “I have decided to get the HPV vaccine,”“I am unsure about getting the HPV vaccine,” and “I have decided not to get the HPV vaccine.” Intentions were assessed using a 4-item measure, with response options ranging from *very unlikely* to *very likely*. This covered participants’ intention within the next 6 mo to find out more information about the HPV vaccine, discuss the HPV vaccine with their health provider, and receive a dose of the HPV vaccine.^22^

Knowledge about HPV was measured using the 13-item Mid-adult Human Papillomavirus Knowledge Scale.^23^ Three additional questions were asked to assess their knowledge on FDA’s approval of the vaccine for mid-adults, scientific evidence on HPV vaccination, and protection provided by HPV vaccination. Participants were expected to indicate either “true,”“false,” or “don’t know” for each statement. Knowledge was then computed as 1-point per correct response to a statement and 0 points for an incorrect or “don’t know” response. The Knowledge scale had good internal consistency (Cronbach’s alpha = 0.78).

Perceived expectation was assessed by asking the participant’s agreement on a 5-point Likert scale with the following statement: “The effectiveness of the HPV vaccine will vary from person to person.” This secondary outcome was selected due to the varied individual benefits of the HPV vaccination in the mid-adult age range.

An 11-item measure was adapted from the Decision Self-efficacy Scale^24^ to assess decisional self-efficacy among participants. The Decision Self-Efficacy Scale evaluates an individual’s self-confidence to make decisions on their own or with support from other sources. We measured participants’ level of confidence in getting information, asking questions, and making decisions about the HPV vaccine. Participants responded using a scale (0: *not all confident* to 4: *very confident*). Decisional Self-Efficacy items had excellent internal consistency (Cronbach’s alpha = 0.96).

Perceived risks of HPV infection were also assessed, including participants’ perceptions of getting an HPV-associated infection, genital/anal warts, or cancer in their lifetime. Response options ranged from *very unlikely* to *very likely*.

For the intervention groups, measures assessed decision-making outcomes based on their perceptions of the decision aid tool.^25^ These measures included rating of information (1 = *poor* to 4 = *excellent*), length of information (3 = *too long* to 1 = *just right*), amount of information (3 = *too much information* to 1 = *just right*), information balance (3 = *slanted towards HPV vaccination* to 1 = *balanced*), and trustworthiness (0 = *not at all* to 1 = *completely*). Furthermore, we assessed participants’ preferences for implementation of the decision tool. This included point of access, ideal time, ideal mode, acceptable provider to discuss the decision aid tool, and likelihoods of providing their contact information in the tool, printing a summary of the content, and taking a screenshot of the content.

### Data Analysis

Descriptive statistics were calculated for demographic characteristics in the overall sample and stratified by experimental group. Pretest scores were computed for the groups that received the pretest. Total cell size and overall proportion were computed for categorical variables, and means and standard deviations were calculated for quantitative variables. Chi-square tests of independence and analysis of variance were conducted to determine any significant differences in demographic characteristics across experimental groups.

Data analysis was carried out in 2 steps for each outcome. The first step involved testing for pretest sensitization due to the Solomon experimental design. Individuals were assigned to 2 factors, with factor A being Treatment v. Control and factor B being Pretest v. No Pretest. Pretest sensitization is indicated if there is a significant interaction between factors A and B on the posttest measures.^26,27^ For continuous outcomes, these interaction terms were assessed via linear regression and, for categorical outcomes, proportional odds logistic regression for ordered categorical variables (Decision, Perceived Risks).

The second step gathered evidence of treatment effect across the 4 experimental groups in the case of no pretest sensitization. Braver and Braver^26^ described a meta-analytic procedure to achieve the largest statistical power in the Solomon design in which groups 1 and 2 are tested for treatment effects controlling for pretest scores while groups 3 and 4 are tested for treatment effects in a posttest-only analysis. These tests were carried out by linear models for continuous outcomes, multinomial regression for the Decision outcome, or proportional odds logistic regression for the Perceived Risk outcomes. The estimates were then meta-analyzed, ensuring that pretest information is controlled for when available and that all participants are included in the final inference. In our analyses, fixed-effects inverse-variance weighting was used to pool the linear or logistic coefficient estimates, calculate pooled confidence intervals (CIs) with the appropriate standard errors, and determine the statistical significance of a pooled test statistic.^28^ Logistic coefficients and CIs were exponentiated to reflect odds ratios (ORs). These effects are interpreted in the same manner as unstandardized regression coefficients or ORs from a single regression model.

## Results

### Group Descriptive Statistics

All descriptive statistics are presented in Table 1. There were no significant differences between the groups on any of the demographic characteristics, consistent with successful random assignment of the groups. Across all pretest measures, there were only 2 domains with significant differences between groups 1 and 2. The groups differed on the Expectation item, in which the control group scored 0.29 points higher on average regarding agreement that HPV vaccine effectiveness varies from person to person (*t* = −2.69, *P* = 0.008). In addition, the control group with pretest reported higher Perceived Risk than the intervention group with pretest across the 3 perceived risk items (HPV-related cancer item: *P* = 0.015; HPV infection item: *P* = 0.001; genital or anal warts item: *P* = 0.003). However, given that the family of pretest comparisons consists of 28 total variables tested, it is not surprising that some differences were detected by chance at the 0.05 level for each test, even if randomization was successful.

**Table 1:** Description of a Sample of US Adults Aged 27 to 45 y by Study Condition Group (*N* = 632)^a^

	Total	Intervention		Control	
		Group 1	Group 3	Group 2	Group 4
Age, y	38.0 (5.0)	37.7 (4.9)	38.3 (4.7)	38.2 (5.0)	37.7 (5.4)
Sex					
Female	312 [0.49]	70 [0.50]	84 [0.49]	74 [0.48]	84 [0.50]
Male	320 [0.51]	70 [0.50]	86 [0.51]	79 [0.52]	85 [0.50]
Gender identity					
Female	310 [0.49]	68 [0.49]	84 [0.49]	74 [0.48]	84 [0.50]
Male	316 [0.50]	69 [0.49]	86 [0.51]	77 [0.50]	84 [0.50]
Other	6 [0.01]	3 [0.02]	0 [0]	2 [0.01]	1 [< 0.01]
Education					
High school or less	246 [0.39]	55 [0.39]	62 [0.37]	63 [0.41]	66 [0.39]
Some college	215 [0.34]	48 [0.34]	56 [0.33]	55 [0.36]	56 [0.33]
College graduate	164 [0.26]	37 [0.26]	50 [0.29]	33 [0.22]	44 [0.26]
Other/missing	7 [0.01]	0 [0]	2 [0.01]	2 [0.01]	3 [0.02]
Health insurance					
Insured	551 [0.87]	124 [0.89]	155 [0.91]	135 [0.88]	137 [0.81]
Uninsured	66 [0.10]	15 [0.11]	10 [0.06]	16 [0.11]	25 [0.15]
Missing/prefer not to answer	15 [0.02]	1 [<0.01]	5 [0.03]	2 [0.01]	7 [0.04]
Race					
Asian	18 [0.03]	5 [0.04]	4 [0.02]	4 [0.02]	5 [0.03]
Black	103 [0.16]	20 [0.14]	31 [0.18]	23 [0.15]	29 [0.17]
White	472 [0.75]	108 [0.77]	128 [0.75]	112 [0.73]	125 [0.74]
Other	38 [0.06]	7 [0.05]	7 [0.04]	14 [0.09]	10 [0.06]
Ethnicity					
Hispanic	95 [0.15]	16 [0.11]	25 [0.15]	24 [0.16]	30 [0.18]
Not Hispanic	534 [0.85]	123 [0.88]	144 [0.85]	128 [0.84]	139 [0.82]
Prefer not to answer	3 [<0.01]	1 [<0.01]	1 [<0.01]	1 [<0.01]	0 [0]
Sexual orientation					
Straight	546 [0.86]	123 [0.88]	156 [0.92]	124 [0.81]	143 [0.85]
LGBQ+	79 [0.13]	16 [0.11]	14 [0.08]	25 [0.16]	24 [0.14]
NA/prefer not to answer	7 [0.01]	1 [<0.01]	0 [0]	4 [0.03]	2 [0.01]
Employment					
Employed	395 [0.53]	88 [0.63]	114 [0.67]	91 [0.60]	102 [0.60]
Not employed	98 [0.16]	25 [0.18]	21 [0.12]	27 [0.18]	25 [0.15]
Other	139 [0.22]	27 [0.19]	35 [0.21]	35 [0.22]	42 [0.25]
Income					
Comfortable	165 [0.26]	41 [0.29]	46 [0.27]	38 [0.25]	40 [0.24]
Getting by	207 [0.33]	36 [0.26]	56 [0.33]	50 [0.33]	65 [0.39]
Difficult	136 [0.22]	27 [0.19]	42 [0.25]	38 [0.25]	29 [0.17]
Very difficult	101 [0.16]	31 [0.22]	21 [0.12]	21 [0.14]	28 [0.17]
Prefer not to answer	23 [0.04]	5 [0.04]	5 [0.03]	6 [0.04]	7 [0.04]
Baseline measures					
Decision					
Decided to get vaccine	—	21 [0.15]	—	20 [0.13]	—
Decided not to get vaccine	—	45 [0.32]	—	64 [0.42]	—
Unsure	—	74 [0.53]		69 [0.45]	—
Intentions: how likely to:					
Research HPV vaccine	—	2.90 (1.27)	—	2.88 (1.44)	—
Talk to provider	—	2.89 (1.40)	—	2.97 (1.45)	—
Get first dose	—	2.64 (1.24)	—	2.52 (1.28)	—
Get first dose if advised	—	3.16 (1.38)	—	2.91 (1.40)	
Decisional Conflict	—	35.5 (22.9)	—	30.6 (23.1)	—
Knowledge	—	4.8 (2.88)	—	4.99 (3.09)	—
Expectation	—	3.57 (0.91)	—	3.86 (0.95)	—
Decisional Self-Efficacy	—	70.6 (23.4)	—	70.8 (25.5)	—
Perceived Risk – HPV-related cancer	—	2.79 (1.08)	—	3.09 (1.01)	—
Perceived Risk – HPV infection	—	2.76 (1.12)	—	3.18 (1.13)	—
Perceived Risk – genital or anal warts	—	2.46 (1.07)	—	2.84 (1.13)	—

HPV, human papillomavirus; LGBQ+, lesbian, gay, bisexual, queer, and questioning.

a Quantitative variables are presented as mean (standard deviation); categorical variables are presented as count [proportion].

### Pretest Sensitization

Table 2 presents all results for tests of pretest sensitization. Across all outcomes, only 1 variable showed evidence of pretest sensitization. The third question around perceived risk, which asked, “How likely are you to get anal or genital warts in your lifetime?” showed pretest sensitization. For all other outcomes, there was no evidence of pretest sensitization, as no interaction between factor A and factor B was significant. For quantitative outcome variables, we report the regression coefficient for the interaction term, the 95% CI, and its *P* value. For categorical outcomes, we report the interaction effect of OR, the 95% CI, and its *P* value. This result indicates that any effect of being in the treatment or control group on the posttest outcome is not modified by the administration of the pretest, except for risk perception of genital or anal warts.

**Table 2:** Tests of Pretest Sensitization among a Sample of US Adults Aged 27 to 45 y (*N* = 632)

	Intervention (Treatment v. Control) by Pretest (Pre + Post v. Posttest Only)	95% CI	*P* Value
Primary outcome			
Decisional Conflict (DC) – Total	B = 1.98	(−3.82, 7.77)	0.50
DC – Uncertainty	B = 1.66	(−5.31, 8.63)	0.64
DC – Informed	B = 2.43	(−4.05, 8.91)	0.46
DC – Values Clarity	B = 2.54	(−3.92, 9.01)	0.44
DC – Support	B = 2.32	(−7.81, 12.27)	0.66
DC – Effective Decision	B = 2.09	(−4.23, 8.40)	0.52
Secondary outcomes			
Decision: decide not to get v. unsure	OR = 0.97	(0.46, 2.05)	0.94
Decision: decide to get v. unsure	OR = 0.88	(0.40, 1.91)	0.74
Decision: decide to get v. decide not to get	OR = 0.90	(0.39, 2.07)	0.81
Intentions – Search online to find more about HPV vaccination for yourself	B = −0.12	(−0.54, 0.31)	0.59
Intentions – talk to a health care provider about HPV vaccination	B = −0.39	(−0.81, 0.03)	0.072
Intentions – get the first dose of the HPV vaccine	B = −0.27	(−0.70, 0.17)	0.23
Intentions – get the first dose of the HPV vaccine if a doctor recommends it to you	B = −0.13	(−0.57, 0.32)	0.58
Knowledge	B = −0.66	(−1.62, 0.31)	0.192
Expectations	B = −0.18	(−0.47, 0.11)	0.23
Decisional Self-Efficacy	B = 4.95	(−2.49, 12.38)	0.192
Perceived Risk – HPV-related cancer	OR = 0.57	(0.32, 1.01)	0.051
Perceived Risk – HPV infection	OR = 0.63	(0.36, 1.11)	0.107
Perceived Risk – genital or anal warts	OR = 0.41	(0.23, 0.72)	0.002

CI, confidence interval; HPV, human papillomavirus; OR, odds ratios of the interaction effect in logistic regression for categorical outcomes.

### Intervention Effectiveness

Evidence of an intervention effect was found for 10 of the 17 outcomes tested, and all meta-analyses of intervention effects are presented in Table 3. Of the primary outcomes, there was a significant intervention effect on the decisional conflict total score as well as all decisional conflict subscales except for uncertainty. The intervention group had lower decisional conflict scores by about 3.6 points, on average. The subscales with the largest reduction in decisional conflict due to intervention were the Support (5.6 points lower) and Values Clarity (4.9 points lower) subscales.

**Table 3:** Effect of Intervention on HPV Vaccine Decision-Making Outcomes among a Sample of US Adults Aged 27 to 45 y (*N* = 632)

	Pooled Estimate	95% CI	Meta-Analytic *P* Value
Primary outcome			
Decisional Conflict (DC) – Total	B = −3.58	(−6.20, −0.97)	0.007
DC – Uncertainty	B = −2.63	(−5.76, 0.51)	0.100
DC – Informed	B = −3.28	(−6.33, −0.23)	0.035
DC – Values Clarity	B = −4.90	(−7.93, −1.87)	0.002
DC – Support	B = −5.61	(−10.14, −1.07)	0.015
DC – Effective Decision	B = −3.12	(−5.97, −0.27)	0.032
Secondary outcomes			
Decision: decide not to get v. unsure	OR = 0.75	(0.50, 1.13)	0.167
Decision: decide to get v. unsure	OR = 1.20	(0.80, 1.81)	0.38
Decision: decide to get v. decide to not get	OR = 1.62	(1.02, 2.58)	0.041
Intentions – search online to find more about HPV vaccination for yourself	B = 0.18	(−0.01, 0.37)	0.069
Intentions – talk to a health care provider about HPV vaccination	B = 0.32	(0.14, 0.51)	<0.001
Intentions – get the first dose of the HPV vaccine	B = 0.20	(0.001, 0.39)	0.049
Intentions – get the first dose of the HPV vaccine if a doctor recommends it to you	B = 0.19	(−0.01, 0.39)	0.067
Knowledge	B = 0.48	(0.07, 0.89)	0.020
Expectation	B = −0.10	(−0.23, 0.34)	0.144
Decisional Self-Efficacy	B = 3.28	(0.10, 6.47)	0.043
Perceived Risk – HPV-related cancer	OR = 0.99	(0.74, 1.32)	0.93
Perceived Risk – HPV infection	OR = 1.05	(0.79, 1.40)	0.73
Perceived Risk – genital or anal warts	OR = 1.22	(0.91, 1.62)	0.183

CI, confidence interval; HPV, human papillomavirus; OR, odds ratio.

Regarding secondary outcomes, those receiving the intervention showed a significant increase in knowledge, greater intention to receive the vaccine and discuss the vaccine with their provider, and greater self-efficacy in their decision making about the HPV vaccine (see Table 3). The intervention was associated with higher odds of deciding to receive the vaccine compared with deciding not to receive the vaccine (OR = 1.62, 95% CI = 1.02, 2.56). The intervention was not associated with higher or lower odds of making a definitive decision on the vaccine compared with being unsure (decide not to get v. unsure: OR = 0.75, 95% CI = 0.50, 1.13; decide to get v. unsure: OR = 1.20, 95% CI = 0.80, 1.81). The intervention did not significantly alter the perceived risks of HPV infection, indicating that exposure to the decision tool did not inadvertently heighten fears around HPV infection and its consequences. Overall, the intervention was perceived more favorably than the control condition in terms of information shared, amount of information, and trustworthiness (Table 4).

**Table 4:** Perspectives about Intervention and Control Conditions among a Sample of US Adults Aged 27 to 45 y (*N* = 632)

	Intervention	Control	*P* Value
Rating of information,^a^ $\bar{x}$ (*s*)			
Information provided about HPV	3.30 (0.74)	3.01 (0.81)	<0.001
Information provided about HPV vaccination	3.29 (0.76)	3.07 (0.81)	<0.001
Information provided about your options	3.25 (0.76)	3.00 (0.86)	<0.001
Action steps	3.22 (0.80)	2.98 (0.84)	<0.001
Time to read, *n* (%)			
Too long	63 (20%)	61 (19%)	0.44
Too short	28 (9%)	39 (12%)	
Just right	219 (71%)	222 (69%)	
Amount of information, *n* (%)			
Too much	44 (14%)	37 (12%)	0.002
Too little	35 (11%)	70 (22%)	
Just right	231 (75%)	215 (67%)	
Balance, *n* (%)			
Slanted toward HPV vaccination	120 (39%)	100 (31%)	0.055
Slanted against HPV vaccination	17 (6%)	29 (9%)	
Balanced	173 (56%)	193 (60%)	
Trust,^b^ $\bar{x}$ (*s*)	2.69 (0.98)	2.36 (1.08)	<0.001

HPV, human papillomavirus.

a Scale 1 to 4 with 1 = *poor*, 4 = *excellent*.

b Scale 0 to 4 with 0 = *not at all*, 4 = *completely trust*.

### Implementation Preferences

The intervention group received additional questions about their preferences for using this decision tool (Table 5). Most preferred to receive the decision tool from a health care provider (55.4%) or look it up online (29.5%). The most acceptable provider to receive this decision tool was a doctor (86.6%), followed by a nurse (48.0%). Among those preferring the health care provider, most wanted it prior to a scheduled visit (42.4%) or any time (30.9%). The ideal delivery mode from a health care provider was by e-mail (35.8%) or patient portal (30.9%). The final page of the tool included a summary of the decision, including inputs made by the user (e.g., values related to HPV vaccination). Participants were questioned about possible ways to share this personalized information with them. Almost half (48%) were likely to provide their contact information in the tool so results could be sent directly to them. Similarly, almost half (47%) would likely take a screenshot of the results screen. Fewer were likely to print the decision summary (35%).

**Table 5:** Decision Tool Implementation Preferences among US Adults Aged 27 to 45 y in the Intervention Group (*n* = 298)

	*n*	%
Preferred mode to access decision tool		
From my health care provider	165	55.4
From my insurance provider	40	13.4
Look it up online	88	29.5
Another option	5	1.7
Health care setting – ideal time (*n* = 165)		
Sent any time	51	30.9
Prior to a scheduled visit	70	42.4
In the waiting room	10	6.1
In the exam room	34	20.6
Health care setting – ideal mode (*n* = 165)		
Sent by text message	29	17.6
Sent by e-mail	59	35.8
Sent by patient portal	51	30.9
On a tablet in the office	19	11.5
On a QR code in the office	7	4.2
Likelihood to provide contact information on decision tool		
Very likely	80	26.8
Likely	63	21.1
50-50 chance	93	31.2
Unlikely	32	10.7
Very unlikely	30	10.1
Likelihood to print the decision tool		
Very likely	60	20.1
Likely	45	15.1
50-50 chance	91	30.5
Unlikely	50	16.8
Very unlikely	52	17.4
Likelihood to screenshot the decision tool		
Very likely	70	23.5
Likely	72	24.2
50-50 chance	85	28.5
Unlikely	34	11.4
Very unlikely	37	12.4
Acceptable provider to talk about the decision tool		
Doctor	258	86.6
Nurse	143	48.0
Medical assistant	89	29.9
Pharmacist	65	21.8
Dentists	16	5.4
Community health worker	50	16.8

## Discussion

The HPV DECIDE tool was developed to assist with the decision-making process for HPV vaccination among mid-adults. After an iterative development process with mid-adult and health care provider input, the decision tool was tested in a randomized control trial. The goal was not to increase HPV vaccine intentions but rather to reduce decisional conflict and improve decisional outcomes. Overall, the HPV DECIDE tool was efficacious in reducing decisional conflict using the overall scale and subscales as well as improving knowledge and decision self-efficacy. Moreover, the decision tool was acceptable to the participants who also provided feedback on modes of delivery in a real-world setting.

As hypothesized, participants who received the decision tool compared with other control conditions had lower decisional conflict as reflected in the total scale score, in addition to the Information, Values, Support, and Effective Decisions subscores. These findings demonstrate that the decision tool helped to make the decision to get vaccinated explicit, provided useful information about the options, and helped to clarify personal values. The effect size (e.g., an average reduction of 3.58 on the total decimal conflict score) was consistent with similar tools informed by the Ottawa Decision Support Framework.^29^ Findings from secondary outcomes also reflect the utility of the tool in promoting shared clinical decision making with regard to mid-adult HPV vaccination. The use of the tool resulted in increased decisional self-efficacy and higher knowledge. Thus, participants were informed, engaged, and empowered to make a decision. The treatment group had a small increase in intentions to talk to a health care provider about HPV vaccination and, to a lesser degree, to initiate the HPV vaccine series. This is as expected as the tool was not designed to promote HPV vaccination. Similarly, the tool did not increase risk perceptions related to HPV infection. Overall, the outcomes support the concept of shared clinical decision making for HPV vaccination rather than promoting vaccine uptake and thus are appropriately aligned with the ACIP guideline.

Participants who received the HPV DECIDE tool reported that the time to read and length were largely acceptable (∼70% just right). The Web-based delivery of the tool allowed for the user to expand on additional information if desired (e.g., background on HPV) and present information aligned with value priorities. As such, this type of Web-based interface gives the user control of how much information to see and may also contribute to the acceptability, whereas a paper-based version would not have these features. The creation of a Web-based tool can also permit integration with electronic medical record systems and patient portals for ease of use. Moreover, participants had more favorable reactions to the information provided about HPV, HPV vaccination, options, and action steps compared with the vaccine information sheet control condition. Assessing user experiences is essential for determining the acceptability of a vaccination decision tool for future practice,^30^ and this decision tool likely benefited from several rounds of end-user input into the design of the tool. Future studies should examine the acceptability of the decision tool in a real-world setting when there are conflicting priorities and less dedicated time for use.

When analyzing the preferences of US adults for implementing the HPV decision support tool, as detailed in Table 5, participants predominantly (55.4%) preferred receiving the decision tool from a health care provider, with the most acceptable provider to talk about the decision tool being a doctor (86.6%), which emphasizes the trusted role of medical professionals and providers in facilitating health-related decisions.^31^ In addition, a significant proportion (29.5%) of respondents favored accessing the tool online, which aligns with the currently growing reliance on digital health solutions.^32,33^ As such, there may be two potential routes of dissemination of this decision tool to reach different audiences for mid-adults. First, disseminating the decision tool through different health systems and clinics will be appropriate for those mid-adults engaged in health care, particularly gynecological clinics, to reach women. Second, this tool can be disseminated through Web-based platforms in partnership with community and/or government public health organizations to reach persons who are not currently engaged in health care services. It cannot be a one-size-fits-all approach to dissemination.

Comparatively, this tool aligns well with other decision aid tools that underscore the importance of timely and relevant information delivery. Recent studies have shown that decision support tools are effectively integrated into clinical settings when their implementation occurs at critical points of care, such as prior to or during preventive health visits or consultations.^34^ Many participants reported that the ideal time to access the tool is prior to their scheduled visit (42.4%). Incorporating the decision tool prior to patient appointments is particularly effective as it allows patients ample time to review and digest the information, fostering a more informed health care experience. This strategy not only prepares patients to engage more actively in their care discussions but also enhances their understanding and satisfaction with the health care services provided.^35^ By strategically timing the introduction of decision tools to match these key interaction points within the health care system, institutions can maximize the benefits of these tools, supporting both clinical efficiency and patient outcomes.

### Limitations

There were several limitations to this study that should be considered. First, we used a nonprobability sample, so the findings are not necessarily generalizable to the entire target population (i.e., unvaccinated adults aged 27 to 45 y in the United States). The use of an online panel likely introduces sampling biases, particularly around the comfort and acceptability of using Web-based tools for research purposes. Second, while we sought to strengthen internal validity through a randomized Solomon design, all outcomes were assessed with an immediate posttest; thus, we cannot determine how these effects were sustained over time. Future research should explore longer-term effects in a clinical and community environment.

## Conclusions

The HPV DECIDE tool demonstrated efficacy in reducing decisional conflict and improving intermediate outcomes important in making informed decisions about mid-adult HPV vaccination. Therefore, this tool may be used to implement the ACIP shared clinical decision-making recommendation for mid-adult HPV vaccination. The tool is acceptable and rated highly by the priority population. Our findings suggest that the tool can be implemented to promote shared clinical decision making.

## References

[1] Viens LJ Henley SJ Watson M , et al. Human papillomavirus–associated cancers—United States, 2008–2012. MMWR Morb Mortal Wkly Rep. 2016;65(26):661–6. Available from: https://www.jstor.org/stable/24858159 [Accessed 25 May, 2024]. 10.15585/mmwr.mm6526a1 27387669

[2] Markowitz LE Dunne EF Saraiya M , et al. Human papillomavirus vaccination. MMWR Recomm Rep [Internet]. 2014;63(5):1–30. Available from: https://www.jstor.org/stable/24832595 [Accessed 25 May, 2024]. 25167164

[3] Meites E Szilagyi PG Chesson HW Unger ER Romero JR Markowitz LE . Human papillomavirus vaccination for adults: updated recommendations of the Advisory Committee on Immunization Practices. MMWR Recomm Rep. 2019;68(32):698–702. DOI: 10.15585/mmwr.mm6832a3 PMC6818701 31415491

[4] Guo Y Bowling J . Human papillomavirus (HPV) vaccination initiation and completion among adult males in the United States. J Am Board Fam Med. 2020;33(4):592–9. DOI: 10.3122/jabfm.2020.04.190464 32675270

[5] Yoo W Koskan A Scotch M Pottinger H Huh WK Helitzer D . Patterns and disparities in human papillomavirus (HPV) vaccine uptake for young female adolescents among U.S. States: NIS-Teen (2008–2016). Cancer Epidemiol Biomarkers Prev. 2020;29(7):1458–67. DOI: 10.1158/1055-9965.epi-19-1103 PMC7415186 32345710

[6] Newman PA Logie CH Lacombe-Duncan A , et al. Parents’ uptake of human papillomavirus vaccines for their children: a systematic review and meta-analysis of observational studies. BMJ Open. 2018;8(4):e019206. DOI: 10.1136/bmjopen-2017-019206 PMC5914890 29678965

[7] US Food and Drug Administration. FDA approves expanded use of Gardasil 9 to include individuals 27 through 45 years old. FDA. Available from: https://www.fda.gov/news-events/press-announcements/fda-approves-expanded-use-gardasil-9-include-individuals-27-through-45-years-old [Accessed 28 May, 2024].

[8] Wheeler CM Skinner SR Del Rosario-Raymundo MR , et al. Efficacy, safety, and immunogenicity of the human papillomavirus 16/18 AS04-adjuvanted vaccine in women older than 25 years: 7-year follow-up of the phase 3, double-blind, randomised controlled VIVIANE study. Lancet Infect Dis. 2016;16(10):1154–68. DOI: 10.1016/s1473-3099(16)30120-7 27373900

[9] Giuliano AR Isaacs-Soriano K Torres BN , et al. Immunogenicity and safety of Gardasil among mid-adult aged men (27–45 years)—the MAM study. Vaccine. 2015;33(42):5640–6. DOI: 10.1016/j.vaccine.2015.08.072 26343499

[10] Gidengil CA Parker AM Markowitz LE , et al. Health care provider knowledge around shared clinical decision-making regarding HPV vaccination of adults aged 27–45 years in the United States. Vaccine. 2023;41(16):2650–5. Available from: https://pubmed.ncbi.nlm.nih.gov/36990828/ [Accessed 25 May, 2024]. 10.1016/j.vaccine.2023.02.051 PMC10337214 36990828

[11] Hurley LP O’Leary ST Markowitz LE , et al. US primary care physicians’ viewpoints on HPV vaccination for adults 27 to 45 years. J Am Board Fam Med. 2021;34(1):162–70. Available from: https://pubmed.ncbi.nlm.nih.gov/33452094/ [Accessed 25 May, 2024]. 10.3122/jabfm.2021.01.200408 PMC10206955 33452094

[12] Thompson EL Wheldon CW Rosen BL Maness SB Kasting ML Massey PM . Awareness and knowledge of HPV and HPV vaccination among adults ages 27–45 years. Vaccine. 2020;38(15):3143–8. DOI: 10.1016/j.vaccine.2020.01.053 32029321

[13] Wheldon CW Garg A Galvin AM Moore JD Thompson EL . Decision support needs for shared clinical decision-making regarding HPV vaccination among adults 27–45 years of age. Patient Educ Couns. 2021;104(12):3079–85. Available from: https://pubmed.ncbi.nlm.nih.gov/33980398/ [Accessed 25 May, 2024]. 10.1016/j.pec.2021.04.016 33980398

[14] Highet M Jessiman-Perreault G Hilton E Law G Allen-Scott L . Understanding the decision to immunize: insights into the information needs and priorities of people who have utilized an online human papillomavirus (HPV) vaccine decision aid tool. Can J Public Health. 2021;112(2):191–8. DOI: 10.17269/s41997-020-00425-z PMC7571294 33078333

[15] Ottawa Hospital Research Institute. Decision aid summary. Ottawa (Canada): Ottawa Hospital Research Institute. Available from: https://decisionaid.ohri.ca/AZsumm.php?ID=2087 [Accessed 3 October, 2024].

[16] Ottawa Hospital Research Institute. Ottawa decision support framework. Ottawa (Canada): Ottawa Hospital Research Institute. Available from: https://decisionaid.ohri.ca/odsf.html [Accessed 25 May, 2024].

[17] Wheldon CW Grace J Zimet G Daley EM Akpan IN Alkhatib SA Thompson EL . Development and evaluation of a decision aid for HPV vaccination among adults aged 27–45 years old in the United States. Computers in Biology and Medicine. 2025 Feb 1;185:109557. 10.1016/j.compbiomed.2024.109557 39674069

[18] Centiment. Research services. Available from: https://www.centiment.co/research-services [Accessed 25 May, 2024].

[19] Centers for Disease Control and Prevention. Vaccines & immunizations: HPV (Human Papillomavirus) VIS. US Department of Health & Human Services. Available from: https://www.cdc.gov/vaccines/hcp/vis/vis-statements/hpv.html [Accessed 3 October, 2024].

[20] Ottawa Hospital Research Institute. User manuals for decisional conflict. Ottawa (Canada): Ottawa Hospital Research Institute. Available from: https://decisionaid.ohri.ca/docs/develop/User_Manuals/UM_decisional_conflict.pdf [Accessed 25 May, 2024].

[21] Stacey D Légaré F Lewis K , et al. Decision aids for people facing health treatment or screening decisions. Cochrane Database Syst Rev. 2017;4(4):CD001431. DOI: 10.1002/14651858.cd001431.pub5 PMC6478132 28402085

[22] Thompson EL Garg A Galvin AM Moore JD Kasting ML Wheldon CW . Correlates of HPV vaccination intentions among adults ages 27–45 years old in the U.S. J Community Health. 2021;46(5):893–2. Available from: https://pubmed.ncbi.nlm.nih.gov/33586085/ [Accessed 25 May, 2024]. 10.1007/s10900-021-00968-3 33586085

[23] Garg A Wheldon CW Galvin AM Moore JD Griner SB Thompson EL . The development and psychometric evaluation of the mid-adult human papillomavirus vaccine knowledge scale in the United States. Sex Transm Dis. 2022;49(6):423–8. Available from: https://journals.lww.com/stdjournal/fulltext/2022/06000/the_development_and_psychometric_evaluation_of_the.5.aspx [Accessed 25 May, 2024]. 10.1097/OLQ.0000000000001615 35608097

[24] O'Connor AM . User Manual - Decision Self-Efficacy Scale [document on the Internet]. Ottawa: Ottawa Hospital Research Institute; 1995 [modified 2002; cited 2024 12 16] 4p. Available from: https://decisionaid.ohri.ca/docs/develop/User_Manuals/UM_Decision_SelfEfficacy.pdf.

[25] O'Connor AM Cranney A . User Manual - Acceptability [document on the Internet]. Ottawa: Ottawa Hospital Research Institute; 1996 [modified 2002; cited 2024 12 16] 4p. Available from: https://decisionaid.ohri.ca/docs/develop/User_Manuals/UM_Acceptability.pdf.

[26] Braver MW Braver SL . Statistical treatment of the Solomon four-group design: a meta-analytic approach. Psychol Bull. 1988;104(1):150–4. DOI: 10.1037/0033-2909.104.1.150

[27] Campbell DT Stanley JC Gage NL. Experimental and Quasi-Experimental Designs for Research. Boston (MA): Houghton Mifflin and Company; 1963.

[28] Borenstein M Hedges LV Higgins JP Rothstein HR . A basic introduction to fixed-effect and random-effects models for meta-analysis. Res Synth Methods. 2010;1(2):97–11. 10.1002/jrsm.12 26061376

[29] Hoefel L Lewis KB O’Connor A Stacey D . 20th anniversary update of the Ottawa Decision Support Framework: part 2 subanalysis of a systematic review of patient decision AIDS. Med Decis Making. 2020;40(4):522–39. DOI: 10.1177/0272989x20924645 32522091

[30] Bruel S Leclercq T Ginzarly M Botelho-Nevers E Frappé P Gagneux-Brunon A . Patient decision aid in vaccination: a systematic review of the literature. Expert Rev Vaccines. 2020;19(4):305–11. DOI: 10.1080/14760584.2020.1742111 32163307

[31] Jolles MP Richmond J Thomas KC . Minority patient preferences, barriers, and facilitators for shared decision-making with health care providers in the USA: a systematic review. Patient Educ Couns. 2019;102(7):1251–62. Available from: https://www.sciencedirect.com/science/article/abs/pii/S0738399118306128 [Accessed 25 May, 2024]. 10.1016/j.pec.2019.02.003 30777613

[32] Heponiemi T Kaihlanen AM Kouvonen A Leemann L Taipale S Gluschkoff K . The role of age and digital competence on the use of online health and social care services: a cross-sectional population-based survey. Digital Health. 2022;8:205520762210744. DOI: 10.1177/20552076221074485 PMC8801649 35111333

[33] Graham TA Ali S Avdagovska M Ballermann M . Effects of a web-based patient portal on patient satisfaction and missed appointment rates: survey study. J Med Internet Res. 2020;22(5):e17955. Available from: https://pubmed.ncbi.nlm.nih.gov/32427109/ [Accessed 25 May, 2024]. 10.2196/17955 PMC7267992 32427109

[34] Chen Z Liang N Zhang H , et al. Harnessing the power of clinical decision support systems: challenges and opportunities. Open Heart. 2023;10(2):e002432. Available from: https://openheart.bmj.com/content/10/2/e002432 [Accessed 25 May, 2024]. 10.1136/openhrt-2023-002432 PMC10685930 38016787

[35] Asmar MLE Dharmayat KI Vallejo-Vaz AJ Irwin R Mastellos N . Effect of computerised, knowledge-based, clinical decision support systems on patient-reported and clinical outcomes of patients with chronic disease managed in primary care settings: a systematic review. BMJ Open. 2021;11(12):e054659. Available from: https://bmjopen.bmj.com/content/11/12/e054659 [Accessed 25 May, 2024]. 10.1136/bmjopen-2021-054659 PMC8705223 34937723

